# Enhancement of antibacterial activity in electrospun fibrous membranes based on quaternized chitosan with caffeic acid and berberine chloride for wound dressing applications†

**DOI:** 10.1039/d4ra05114a

**Published:** 2024-10-30

**Authors:** Po-Hsun Chiu, Zhao-Yi Wu, Chih-Chin Hsu, Yung-Chi Chang, Chang-Ming Huang, Cheng-Ti Hu, Che-Min Lin, Shin C. Chang, Hsyue-Jen Hsieh, Chi-An Dai

**Affiliations:** a Department of Chemical Engineering, National Taiwan University Taipei 10617 Taiwan hjhsieh@ntu.edu.tw polymer@ntu.edu.tw; b Graduate Institute of Microbiology, College of Medicine, National Taiwan University Taipei 10051 Taiwan

## Abstract

Electrospun nanofibers made from chitosan are promising materials for surgical wound dressings due to their non-toxicity and biocompatibility. However, the antibacterial activity of chitosan is limited by its poor water solubility under physiological conditions. This study addresses this issue by producing electrospun nanofibers mainly from natural compounds, including chitosan and quaternized chitosan, which enhance both its solubility for electrospinning and the antibacterial activity of the resulting electrospun nanofibers. Additionally, antimicrobial agents like caffeic acid or berberine chloride were incorporated. The glutaraldehyde-treated nanofibers showed improved mechanical properties, with an average tensile strength exceeding 2.7 MPa, comparable to other chitosan-based wound dressings. They also demonstrated enhanced water stability, retaining over 50% of their original weight after one week in phosphate-buffered saline (PBS) at 37 °C. The morphology and performance of these nanofibers were thoroughly examined and discussed. Furthermore, these membranes displayed rapid drug release, indicating potential for inhibiting bacterial growth. Antibacterial assays revealed that S2-CX nanofibers containing caffeic acid were most effective against *E. coli* and *S. aureus*, reducing their survival rates to nearly 0%. Similarly, berberine chloride-containing S4-BX nanofibers reduced the survival rates of *E. coli* and *S. aureus* to 19.82% and 0%, respectively. These findings suggest that electrospun membranes incorporating chitosan and caffeic acid hold significant potential for use in antibacterial wound dressings and drug delivery applications.

## Abstract 2

Composite electrospun membranes composed of quaternized chitosan, chitosan, poly(ethylene oxide) and either caffeic acid or berberine demonstrated improved hydrophilicity and enhanced antibacterial activity against *E. coli* and *S. aureus*.

## Graphical abstract

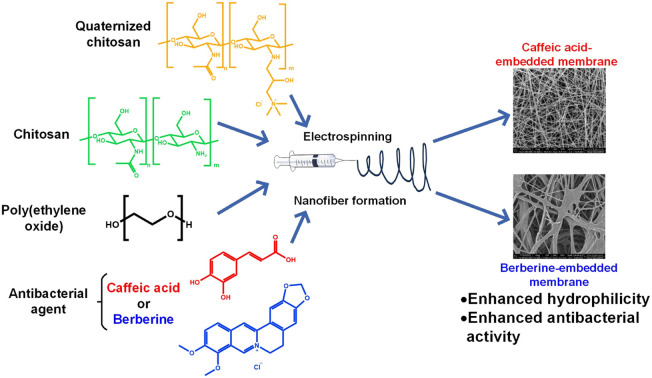

## 1. Introduction

Chitosan, a natural polysaccharide derived from chitin, is a copolymer composed of β(1→4)-linked d-glucosamine and *N*-acetyl-d-glucosamine monomers obtained through partial deacetylation in alkaline conditions from sources such as crustacean shells, insects, and fungal cell walls.^1^ Although chitosan's free base form is not water-soluble, it can dissolve in acidic solutions (pH < 6.5) due to protonation of its amino groups.^2^ The biomedical applications of chitosan stem from its attributes including biocompatibility, biodegradability, non-carcinogenicity, and antibacterial properties.^3–5^

Despite demonstrating promising wound healing properties, the antibacterial mechanism of chitosan remains incompletely understood, potentially hindering its further development for enhanced antibacterial efficacy.^6^ One commonly proposed mechanism involves the disruption of bacterial cell walls or membranes. In acidic solutions, the protonated amino groups of chitosan enable its adsorption onto bacterial surfaces *via* electrostatic interactions, which alter cell permeability and induce membrane lysis.^7^ Other potential mechanisms of chitosan's antibacterial action include DNA interaction, metal ion chelation, and the formation of a polymer layer that hinders nutrient transfer.^8–10^ To further enhance chitosan's solubility and antibacterial efficacy under physiological conditions, researchers have explored chemical modifications targeting the amino and hydroxyl groups present in chitosan.^11^ Common modifications include the introduction of amino, hydroxyl, carboxyalkyl, guanidino, and quaternary alkyl groups.^12–17^ In particular, quaternized chitosan, featuring permanently positive charges from quaternary alkyl groups, exhibits enhanced solubility and antibacterial activity in neutral and basic environments.^18,19^

In order to develop bioactive wound dressings, it is essential that they are designed to mimic the natural structure of human skin.^20^ The primary types of dressings readily accessible on the market today include films, foams, sponges, hydrogels, and nanofiber films. Among these materials, electrospun nanofiber membranes are particularly notable for their significant potential in wound dressings.^21^ Electrospinning produces continuous polymer nanofibers, forming porous membranes resembling the extracellular matrix (ECM), which facilitate cell proliferation and adhesion, therefore, ideal for commercial wound dressings.^22–27^ Despite the difficulty of fabricating chitosan nanofibers *via* electrospinning due to strong hydrogen bonding, adding poly(ethylene oxide) (PEO) was commonly found effective in enhancing electrospinnability and preserving chitosan fiber biocompatibility.^28–33^ For instance, Pakravan *et al.* fabricated defect-free electrospun nanofibers with 90 wt% high chitosan content by incorporating PEO.^34^ While chitosan-based electrospun nanofibers have been extensively documented, only a limited number of studies have investigated the production of chitosan/quaternized chitosan composite electrospun membranes. For instance, Andreica *et al.* developed chitosan/*N*-(2-hydroxy)propyl-3-trimethyl ammonium chitosan chloride (HTCC)/PEO electrospun nanofibers. Pristine nanofibers were then utilized as wound dressings after selectively removing PEO. These nanofibers exhibited significant antibacterial activity against *Escherichia coli* (*E. coli*), with bacterial inhibition rates >99%, following a 6 hour incubation. Nevertheless, these HTCC-containing nanofibers exhibited relatively lower water stability compared to pure chitosan nanofibers, attributed to the increased water solubility of HTCC. Furthermore, there was still room for improvement in the antibacterial activity of the nanofibers against *Staphylococcus aureus* (*S. aureus*).^35^

Natural compounds renowned for their potent antibacterial properties are gaining recognition in wound dressings for infection prevention and healing recently. For instance, caffeic acid, derived from fruits and coffee beans, serves as a widely-used antimicrobial agent with potential in cancer and inflammation drug development. Similarly, berberine, extracted from the genus *Berberis*, demonstrates antineoplastic and antibacterial effects by impeding cancer cell growth and bacterial protein synthesis.^36^ Nevertheless, both compounds encounter limitations such as low water solubility and poor bioavailability, constraining their use in biomedical applications.^37,38^ To address these drawbacks, the incorporation of caffeic acid and berberine into nanofibers *via* electrospinning has been adopted as a strategy to enhance water solubility and antibacterial activity. Narayanan *et al.* prepared poly(vinyl alcohol) (PVA) electrospun nanofibers loaded with complexes of caffeic acid and cyclodextrins to boost the solubility and antibacterial activity of caffeic acid.^39^ In addition, Zhang *et al.* fabricated sodium alginate (SA)/PVA/PEO/berberine chloride composite electrospun microfiber membranes. These membranes, with 5 wt% berberine chloride, demonstrated enhanced antibacterial activity against *E. coli* with 97% antibacterial efficiency.^40^

Based on the literature data, our research aims to create electrospun membranes that incorporate chitosan, quaternized chitosan, and natural antibacterial compounds like caffeic acid or berberine chloride. The objective is to produce novel biocompatible and highly antibacterial multifunctional biomaterials suitable for the development of advanced wound dressings with superior performance. In this study, composite nanofiber mats containing four primary components – chitosan, lab-synthesized quaternized chitosan, PEO, and either caffeic acid or berberine chloride – are fabricated using single-needle electrospinning technique. Furthermore, to improve the water stability of these highly water-soluble nanofibers and elevate their mechanical properties, vapor-phase crosslinking with glutaraldehyde is conducted. The characteristics of these nanofibers, including morphology, mechanical properties, drug release behavior, water stability, and antibacterial activity against *E. coli* and *S. aureus*, are thoroughly investigated and analyzed in our study. Finally, antibacterial activity tests are conducted on the membranes against both Gram-negative *E. coli* and Gram-positive *S. aureus* bacteria, demonstrating their exceptional antibacterial properties and potential application in wound dressing materials.

## 2. Experimental section

### 2.1. Materials

Chitosan (MW ≅ 250 000, degree of deacetylation (DD) = 91.7%) was procured from Kiotek Co. (Taipei, Taiwan). Glycidyltrimethylammonium chloride (GTMAC) and 50 wt% aqueous glutaraldehyde (GA) solution were obtained from Tokyo Chemical Industry Co. (Japan). Methanol was sourced from Macron Fine Chemicals (USA), while 95% v/v ethanol was acquired from Taiwan Tobacco and Liquor Co. (Taiwan). Acetone was purchased from TEDIA (USA), and sodium chloride from Honeywell Fluka (USA). 2′,7′-Dichlorofluorescein was obtained from Alfa Aesar (UK), and dextrin from Acros Organics (USA). Fisher Scientific (UK) provided the silver nitrate aqueous solution (0.1 M). Caffeic acid (product no. C0625) and berberine chloride (product no. B3251), along with deuterium oxide, acetic acid-d_4_, gelatin (from bovine skin), and poly(ethylene oxide), were all sourced from Sigma-Aldrich (USA). Acetic acid was acquired from J.T. Baker (USA). Luria broth (LB) was procured from BioShop (Canada), and brain heart infusion broth (BHI) from Biolife (Italy). Gram-negative *Escherichia coli* (*E. coli*, strain ATCC 25922) and Gram-positive *Staphylococcus aureus* (*S. aureus*, strain JE2) were utilized in bactericidal experiments.

### 2.2. Synthesis of *N*-[(2-hydroxyl)propyl-3-trimethylammonium] chitosan chloride (HTCC)

HTCC was synthesized following a modified method developed by Lim *et al.*,^41^ Cho *et al.*,^42^ and Gorshkova *et al.*^43^ Initially, 70 ml of ultrapure water was poured into a three-necked bottle, followed by slow addition of 6 g (0.024 mmol) of chitosan powder, thoroughly stirred with a stir bar. The suspension was heated to 85 °C, and then 21.857 ml of 80 wt% GTMAC (0.136 mol) aqueous solution was added dropwise through an addition funnel. Additional water (70 ml) was introduced to reduce viscosity. The reaction continued at 85 °C for 6 hours. Water-soluble HTCC was then precipitated by centrifuging the reaction mixture. The clear supernatant was combined with acetone to precipitate the white HTCC crude product, stored overnight at 4 °C, and then filtered and rinsed with methanol and ethanol/acetone mixed solution (v/v = 4/1) three times. Vacuum filtration collected the product, which was then cut into small pieces with surgical scissors. The purified HTCC was dried at 65 °C for 12 hours and stored for further experiments.

### 2.3. ^1^H nuclear magnetic resonance

The nuclear magnetic resonance (NMR) spectra of the HTCC sample dissolved in a 20 wt% solution of acetic acid-d_4_/deuterium oxide was recorded using a 500 MHz NMR spectrometer (Bruker AVIII-500) at the Instrument Center of National Taiwan University.

### 2.4. Degree of substitution of HTCC

The degree of substitution of HTCC was determined using conductimetric titration,^44,45^ employing a standard solution of silver nitrate. Briefly, 0.1 g HTCC was dissolved in 100 ml of deionized water to prepare the HTCC solution, to which the 0.017 M AgNO_3(aq)_ solution was added dropwise. The change in conductivity of the HTCC solution was monitored using a portable conductivity meter (COND6 plus, Eutech). The degree of substitution (DS), which was derived from the concept of number-average fraction of amino groups quaternized,^45^ was calculated using the following eqn (1):1
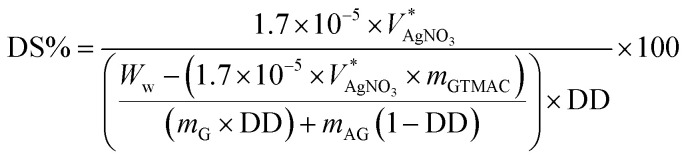
where 
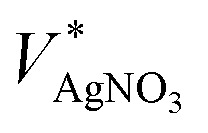
 denotes the equivalent volume of titration, signifying the volume of AgNO_3(aq)_ solution added when the conductivity of the titrated solution reaches its lowest value. In the denominator, *W*_w_ represents the weight of HTCC (0.1 g), *m*_GTMAC_ stands for the molecular weight of GTMAC (MW = 151.63), *m*_G_ represents the molecular weight of glucosamine repeating unit in chitosan (MW = 161), *m*_AG_ indicates the molecular weight of the acetyl glucosamine repeating unit in chitosan (MW = 203) and DD signifies the degree of deacetylation of chitosan (DD = 91.7%).

### 2.5. Preparation of composite electrospinning solution and electrospun fibrous membranes

The method for fabricating electrospun membranes was adapted from Kuo's protocol.^46^ First, to prepare the electrospinning solutions, chitosan PEO, HTCC, and either caffeic acid or berberine chloride were sequentially added to a 20 wt% acetic acid aqueous solution. The mixture was then thoroughly mechanically stirred for 2 hours to ensure complete dissolution of the solutes. Table 1 presents the codes and compositions of the different electrospinning solutions.

**Table 1:** Compositions of dried electrospun fibrous membranes

Code	Chitosan (wt%)	HTCC (wt%)	PEO (wt%)	Caffeic acid (wt%)	Berberine chloride (wt%)	GA vapor-crosslinking time (hour)
aS1-bC^c^U	76.9	—	7.7	15.4	—	0
S1-CX	76.9	—	7.7	15.4	—	1
S2-CU	38.5	38.5	7.7	15.4	—	0
S2-CX	38.5	38.5	7.7	15.4	—	1
S2-CX2	38.5	38.5	7.7	15.4	—	2
S2-CX3	38.5	38.5	7.7	15.4	—	3
S3-BU	76.9	—	7.7	—	15.4	0
S3-BX	76.9	—	7.7	—	15.4	1
S4-BU	38.5	38.5	7.7	—	15.4	0
S4-BX	38.5	38.5	7.7	—	15.4	1
S4-BX2	38.5	38.5	7.7	—	15.4	2
S4-BX3	38.5	38.5	7.7	—	15.4	3
S5-0X	91	—	9	—	—	1
S6-0X	45.4	45.4	9.1	—	—	1

a S1 to S6 represent chitosan-based fibrous membranes with different composition. S1 and S2 denote membranes containing caffeic acid and without or with HTCC, respectively. S3 and S4 denote membranes containing berberine chloride and without or with HTCC, respectively. S5 and S6 denote membranes not containing natural compounds and without or with HTCC, respectively.

b “C” denotes caffeic acid. “B” denotes berberine chloride. “0” indicates without natural compounds.

c “U” denotes no GA crosslinking treatment. “X” denotes GA crosslinking treatment, and the number following “X” represents the crosslinking time in hours. If “X” is not followed by a number, it indicates a crosslinking time of 1 hour.

To fabricate the nanofibers, an electrospinning solution was loaded into a plastic syringe connected to a syringe pump. The syringe was then attached to a PTFE microtube fitted with a stainless-steel syringe needle (size: 23 gauge). Electrospinning parameters were set as follows: working voltage of 25 kV, feeding rate of 0.05 ml min^−1^, collecting distance of 15 cm, collector rotation speed of 300 rpm, and ambient chamber temperature at 33 °C.

### 2.6. Scanning electron microscope (SEM)

SEM samples were prepared by using a scalpel to cut several square pieces of membranes, each measuring 0.25 cm^2^. These samples were then affixed to a carbon tape on a carrier. Subsequently, a thin layer of gold was deposited onto the samples using a sputter machine. The coated samples were introduced into the vacuum chamber of an SEM (JSM-5310, JEOL), where the fiber morphology of the membranes was observed at high magnification. The average diameter of the fibers was calculated using an image analysis tool in the Zeiss Smart SEM® software for their quantification.

### 2.7. Glutaraldehyde vapor crosslinking

In brief, membranes and a glass dish filled with 0.5 ml 50 wt% glutaraldehyde aqueous solution were placed in a desiccator. Subsequently, the desiccator was evacuated to vaporize the glutaraldehyde. Once the vapor crosslinking process was completed, the crosslinked membranes were transferred to a vacuum oven for 12 hours to eliminate excess glutaraldehyde from the membranes.

### 2.8. Mechanical properties of membranes

To comprehend the mechanical properties of electrospun fibrous membranes, the membranes were made and cut into dumbbell-shaped pieces. The ultimate tensile strength and elongation at break were assessed using a tensile testing machine (AI-3000, Gotech Testing Machine Inc). The stretching rate was maintained at 10 mm min^−1^.

### 2.9. Membrane stability test in PBS

The membranes underwent pre-drying in an oven to remove residual water. The weight of the dry membrane was then measured and recorded as *W*_i_. Subsequently, pre-dried membranes were placed in centrifuge tubes, and 40 ml of phosphate-buffered saline (PBS) was added to each tube. These tubes, containing membranes immersed in PBS, were placed inside an orbital shaker incubator and maintained at 37 °C with a rotational speed of 60 rpm for the duration of the experiment. At specific intervals, the membrane samples were withdrawn from PBS, and rinsed with deionized water. After rinsing, the membrane samples were placed in a vacuum oven and dried for 12 hours to remove moisture. The membranes were then weighed to determine the residual dry weight, noted as *W*_f_. The percentage of residual weight of the membrane at different dissolution times was calculated using the following eqn (2):2
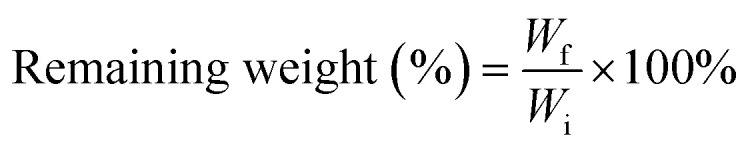


### 2.10. Drug release test

To monitor the concentration of caffeic acid and berberine released from the membranes into PBS over time, we first constructed calibration curves for caffeic acid and berberine. Initially, we identified the characteristic absorption peaks for caffeic acid and B, found at 283 nm and 260 nm, respectively. Subsequently, standard solutions of caffeic acid and B were prepared at concentrations of 2.5, 5, 10, 12.5, 20, 25, 30, 40, and 50 mg l^−1^. The absorbance of these solutions at the designated wavelengths was measured using a UV/VIS spectrophotometer. Finally, calibration curves for caffeic acid at 283 nm and B at 260 nm were plotted based on the concentrations of the standard solutions and their corresponding absorbances. These calibration curves are presented in Fig. S1 and S2, respectively, in the ESI.† For the drug release test, membranes were inserted into bottles, and 150 ml of pH 7.4 PBS was added to each bottle. The bottles were then placed into an orbital shaker incubator at 37 °C and 150 rpm. Samples of 3 ml each were collected at intervals of 2, 5, 10, 20, 30 minutes, 1, 2, 4, 6, 24 hours. Simultaneously, an equal volume of 3 ml of PBS solution was added to compensate for the volume removed. The absorbance of the samples was measured using a UV/VIS spectrophotometer, and the concentrations of the drugs in PBS were determined by fitting the absorbance values into the calibration curves.

### 2.11. *In vitro* antibacterial growth test

The antibacterial test followed the method developed by Lin C. M. *et al.*^47^ Glutaraldehyde-crosslinked electrospun membranes was placed inside a desiccator filled with ethanolamine vapor. The membranes were left in the desiccator for 2 hours to block unreacted aldehyde groups. The ethanolamine-treated membrane was cut into several circular membranes with a diameter of 2 cm. These circular membranes were sterilized by exposure to UV light for 12 hours. Two circular membranes were placed in each well of a 12-well plate. Next, 0.1 ml of diluted bacteria solution (10^5^ CFU ml^−1^) was added to each well, ensuring complete moisture saturation of the circular membranes. Control groups were prepared following the same procedure as the experimental groups, except that the electrospun membranes were omitted. Both experimental and control groups were then placed in an incubator at 37 °C for 20 hours. After the incubation period, the electrospun membranes from each well were transferred to centrifuge tubes containing 10 ml of sterile PBS. The tubes were then vibrated using a vortex mixer to detach the bacteria from the membrane surfaces. The PBS containing bacteria was serially diluted, and 0.025 ml of the diluted bacterial solution was spread onto agar plates for CFU enumeration. The following eqn (3) was used to calculate the bacterial survival ratio (%):3
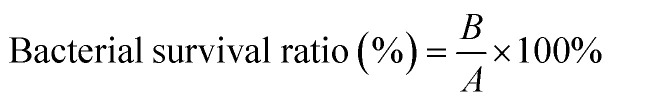
where *A* denotes the number of colonies on the agar plates of the control group and *B* signifies the number of colonies on the agar plates of the experiment group.

### 2.12. Statistical analysis

Experiments were conducted with a minimum of three samples utilized (*n* ≥ 3). Results were expressed as mean of three or more replicates in each experimental group ± standard deviation (SD). Statistical significance was estimated by Student's *t*-test. *P* value less than 0.05 was considered to be statistically significant.

## 3. Results and discussion

### 3.1. Characterization of quaternized chitosan (HTCC)

**Scheme 1:**
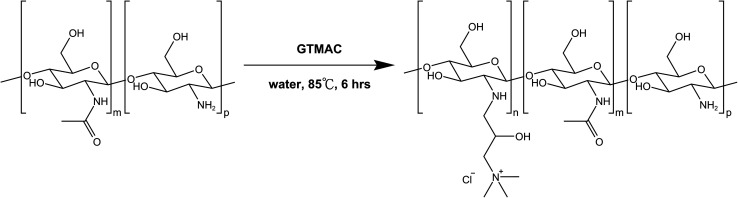
Synthesis route of water-soluble chitosan derivatives—HTCC.

**Fig. 1:**
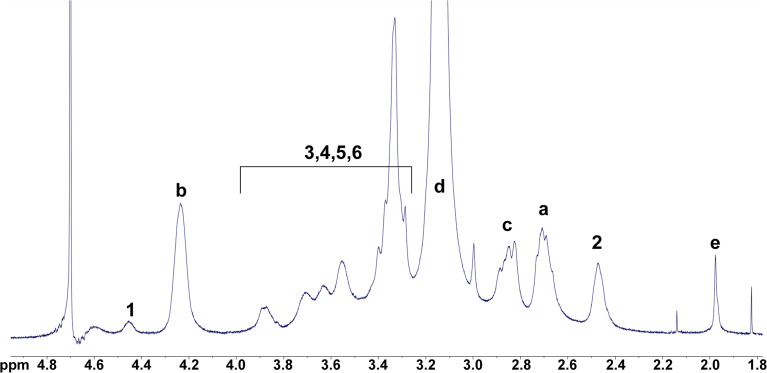
The NMR spectra of HTCC and chitosan with analysis of NMR signals.

The reaction route for the synthesis of HTCC in this study is illustrated in Scheme 1. HTCC was synthesized by reacting GTMAC with chitosan under neutral aqueous conditions.^41^ The ^1^H NMR spectra of the synthesized HTCC are shown in Fig. 1, with the corresponding proton assignments displayed in the inset diagram. The signal peaks labeled 1, 2, 3, 4, 5, and 6 correspond to hydrogen atoms on the synthesized HTCC. The “a” peak at 2.708 ppm represents the signal from two hydrogen atoms near the nitrogen attached directly to chitosan's backbone. The “b” peak at 4.236 ppm corresponds to a single hydrogen atom at the asymmetric center of the GTMAC moieties. The “c” peak at 2.825 ppm arises from two hydrogen atoms located between the asymmetric center and the quaternary amine group. The intense “d” peak at 3.144 ppm is due to signal overlap from nine hydrogen atoms on the three methyl groups of the quaternary amine. Lastly, the “e” peak at 1.977 ppm corresponds to three hydrogen atoms on the acetyl group of the *N*-acetylglucosamine monomers in quaternized chitosan. The ^1^H NMR spectrum confirms that GTMAC reacts with chitosan, becoming part of its side chain.

**Fig. 2:**
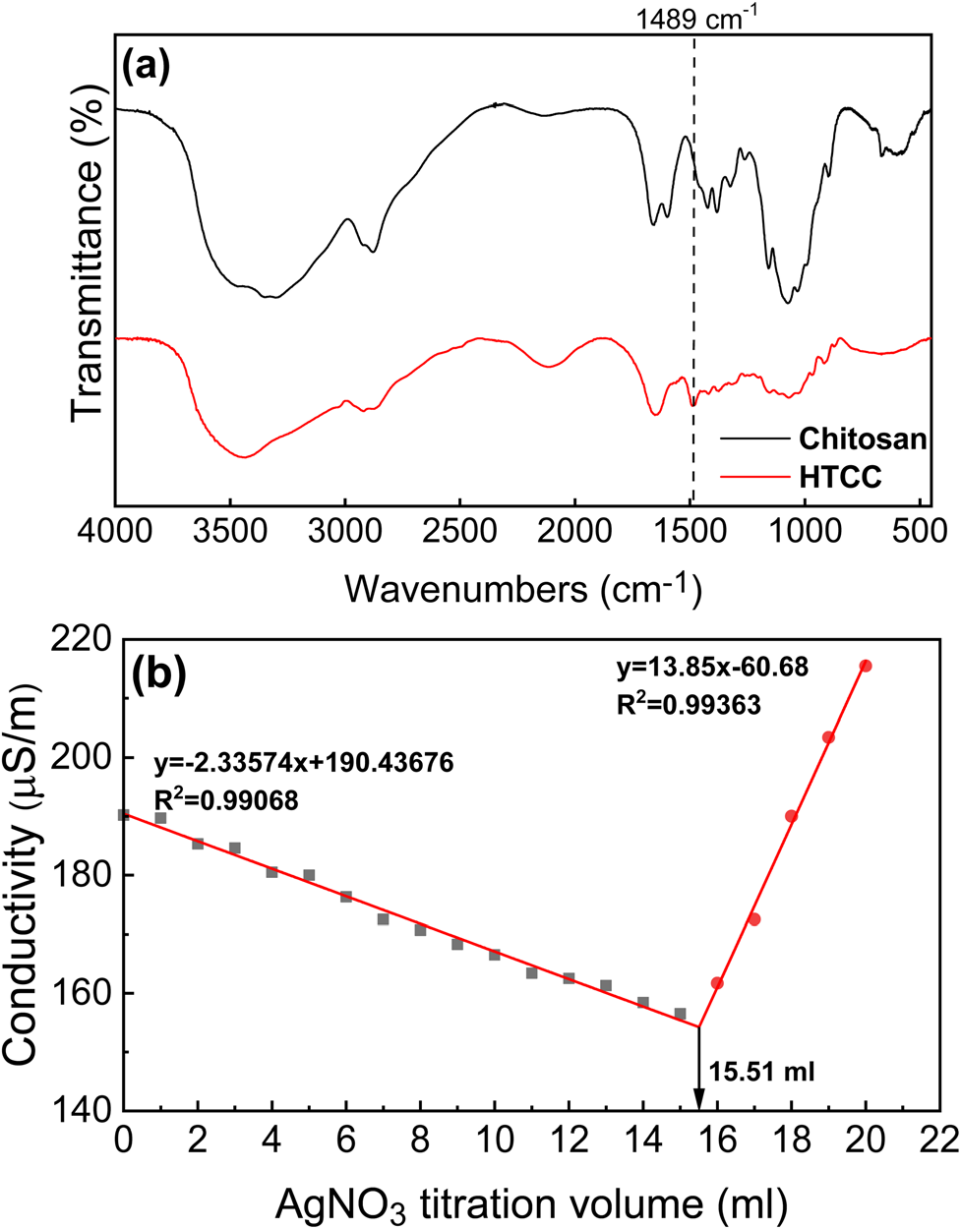
(a) The FT-IR spectra of chitosan and HTCC. (b) The conductometric titration curve for dilute HTCC aqueous solution.

Fig. 2a displays the FTIR spectra of chitosan and HTCC. In the HTCC spectrum, a peak at 1489 cm^−1^ corresponds to the C–H stretching of the methylene group (–CH_2_–), indicating of the presence of 2-hydroxyl propyl-3-trimethylammonium moieties. In contrast, the FTIR spectrum of the pristine chitosan does not show a distinct peak at 1489 cm^−1^. Therefore, the synthesis of HTCC is confirmed by the results obtained from both NMR and FTIR measurements.

Given the presence of chlorine counter ions for the quaternary ammonia in HTCC, using a silver nitrate solution (AgNO_3(aq)_) to titrate the HTCC solution proved to be and effective method for determining the degree of substitution of amino groups on the HTCC polymer chain. When AgNO_3(aq)_ is added to the HTCC aqueous solution, silver ions promptly react with chloride ions to form insoluble white silver chloride (AgCl) precipitate. At the same time, nitrate ions replace chloride ions as the new counterions for HTCC. The reaction formula is as follows:R–N(CH_3_)^+^Cl^−^ + AgNO_3_ → AgCl↓ + R–N(CH_3_)_3_^+^NO_3_^−^

The results of the titration experiment are depicted in Fig. 2b. When HTCC is dissolved in water, the solution displays certain electrical conductivity due to the presence of chloride ions and quaternary ammonium ions. It is evident that the gradual addition of AgNO_3(aq)_ to the HTCC solution led to a decrease in the solution's conductivity, attributed to the formation of AgCl precipitate and a reduction in Cl^−^ concentration. Once all chloride ions in the solution are consumed, the conductivity reaches its minimum, indicating the equivalence volume in the titration. Further addition of AgNO_3(aq)_ to the HTCC solution results in an increase in solution conductivity, attributed to the rising concentrations of Ag^+^ and NO_3_^−^ ions in the solution. Consequently, two linear regression functions can be derived from the titration curves, with the intersection of these functions indicating the equivalent volume of the HTCC solution, measured at 15.51 ml, as illustrated in Fig. 2b. The equivalent volume 
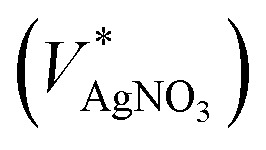
 is then inserted into eqn (1) to compute the degree of amino group substitution (DS) on HTCC, resulting in a value of 78.6%. This result confirms that majority of the amino groups on chitosan react with GTMAC, resulting in improved water solubility due to the introduction of quaternary ammonium moieties. As a result, HTCC shows significant potential for enhancing the antibacterial properties of the thus made electrospun membranes, owing to its high degree of quaternization.^48^

### 3.2. Morphology of electrospun fibrous membrane

**Scheme 2:**
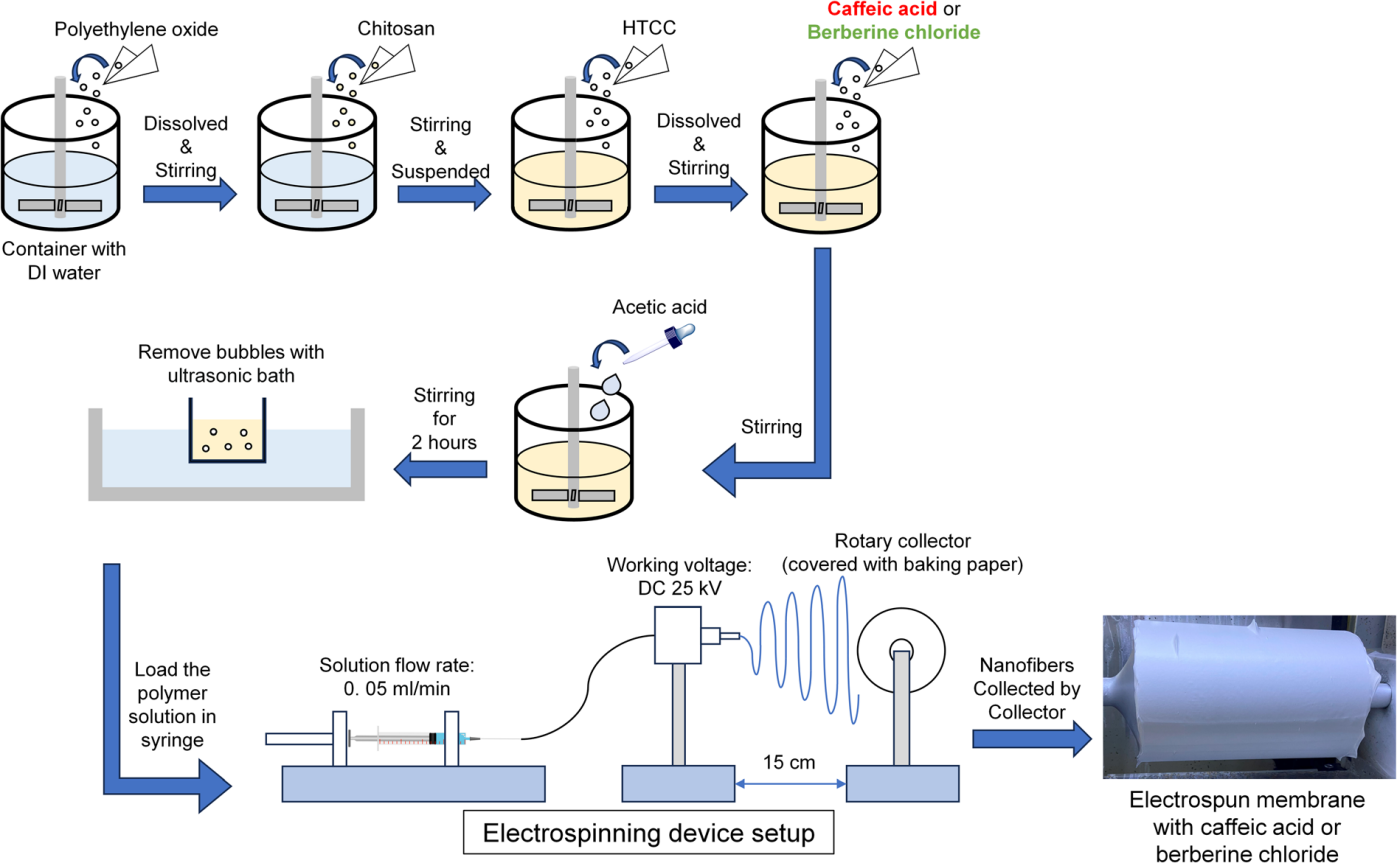
Detail preparation route for electrospun fibrous membranes in this study.

**Fig. 3:**
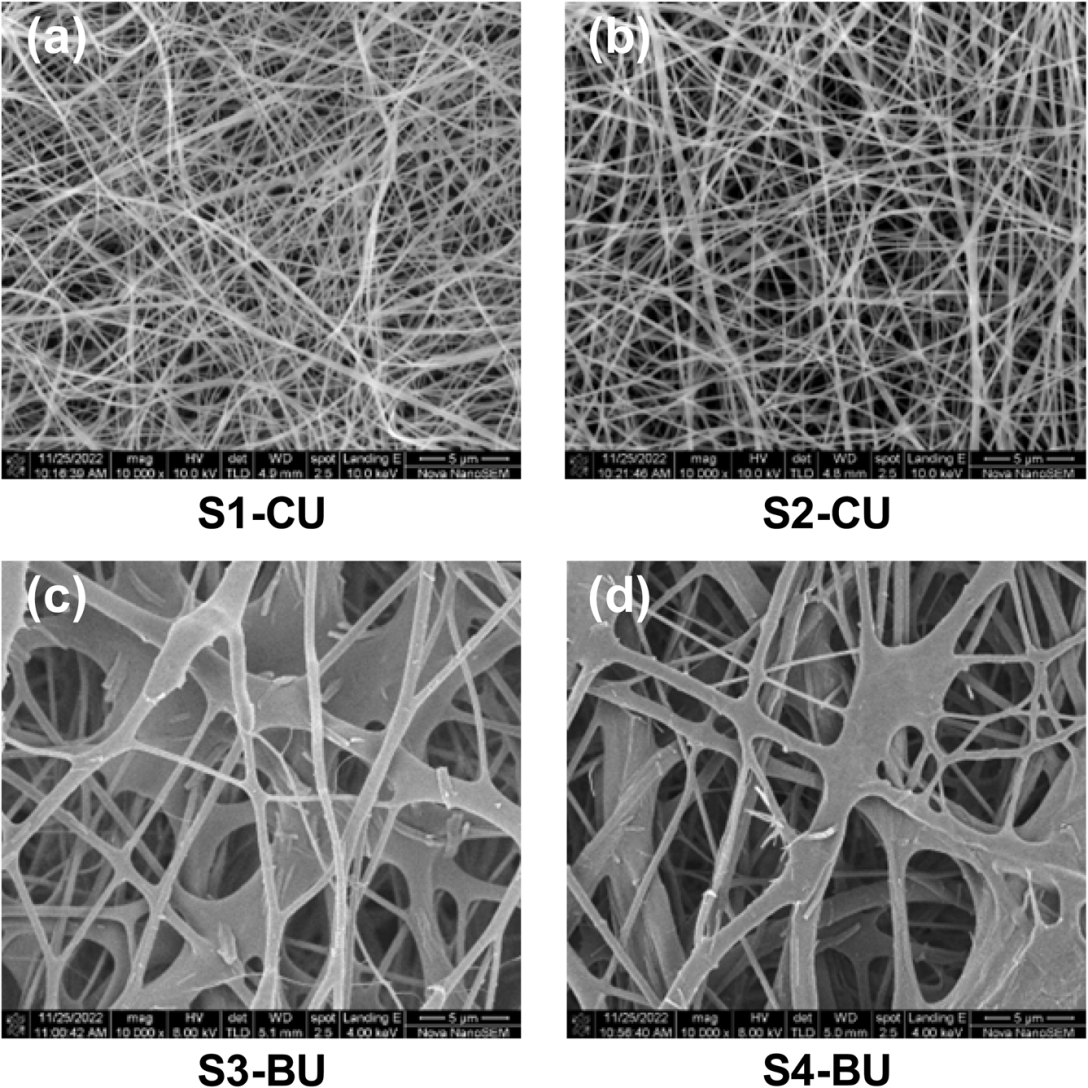
The SEM images of various electrospun fibrous membranes. (a) S1-CU; (b) S2-CU; (c) S3-BU; (d) S4-BU. Scale bar = 5 μm (10 000×).

To fabricate antibacterial wound dressing with a high surface-area-to-volume ratio morphology, we employ electrospinning to prepare chitosan-based electrospun membranes incorporated with caffeic acid or berberine chloride, as illustrated in Scheme 2. The composition of the electrospinning solution is a critical factor influencing the morphology of electrospun nanofibers, as demonstrated by SEM analysis. Fig. 3 presents SEM images of electrospun fibrous membranes, with Fig. 3a and b showing fibers produced from caffeic acid-containing chitosan solutions without (S1-CU) and with (S2-CU) the addition of HTCC, respectively. The images reveal that both S1-CU and S2-CU exhibit similar fiber morphology, characterized by uniform, bead-free nanofibers with average diameters of approximately 326 nm and 371 nm, respectively, as shown in Table 2. This morphology resembles that of the extracellular matrix (ECM) found in all living tissues and organs, which plays a crucial role in promoting cell adhesion, migration, growth, differentiation, and apoptosis. In addition, the interwoven nature of the electrospun nanofibers may enhance drug delivery due to their high surface-to-volume ratio.^49,50^ Therefore, these HTCC and caffeic acid included nanofibers, with their ECM-mimetic structure, hold significant potential for wound healing applications.^51^

**Table 2:** The average fiber diameters of S1-CU, S2-CU, S3-BU and S4-BU

Code	Fiber diameter (nm)
S1-CU	326 ± 86
S2-CU	371 ± 70
S3-BU	438 ± 74
S4-BU	538 ± 82

Fig. 3c and d show images of fibers produced from chitosan solutions containing berberine chloride, both without (S3-BU) and with (S4-BU) the addition of HTCC. The S3-BU and S4-BU membranes exhibit inter-fiber merging and have larger diameters compared to the S1-CU and S2-CU fibers. Specifically, the average diameters of the S3-BU and S4-BU fibers are approximately 440 nm and 540 nm, respectively. This merging likely occurs during electrospinning due to a low volume charge density in the jets, possibly caused by undissolved berberine particles, as berberine chloride has much lower solubility compared to caffeic acid. These particles may cause the fibers to come into contact before the solvent fully evaporates, leading to fusion and inter-fiber merging.^52,53^ Additionally, the low volume charge density can result in larger fiber diameters due to a reduced electrostatic stretching force on the jets.^54,55^ The SEM images in Fig. 3 clearly show that the composition of the polymer solutions significantly affects the resulting nanofiber morphology.

### 3.3. Membrane crosslinking and mechanical property test

To reduce water solubility and maintain the morphology of nanofibers in aqueous environments for subsequent applications, the electrospun membranes are treated with glutaraldehyde vapor for crosslinking. The four types of fibrous membranes described in Section 3.2 are all crosslinked for three different treatment times (1, 2, and 3 hours). Following this treatment, tensile tests are performed on the fibrous membranes. The stress–strain curves of S1-CU, S2-CU, S3-BU, and S4-BU from the tensile testing are shown in Fig. S3 in the ESI.†

**Fig. 4:**
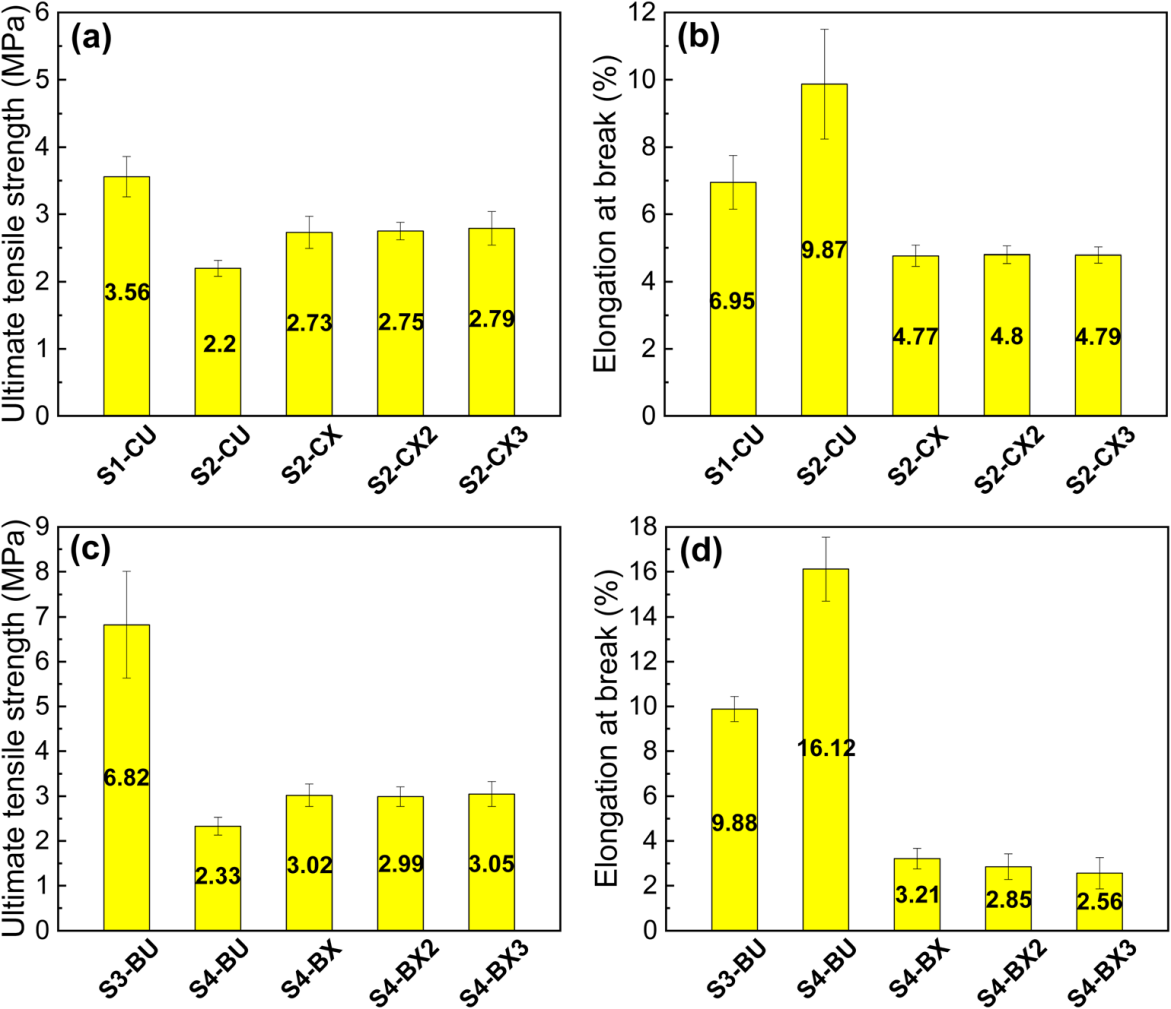
(a) The ultimate tensile strengths and (b) elongation at break of caffeic acid-containing un-crosslinked membranes (S1-CU, S2-CU) and 1 hour, 2 hour, 3 hour crosslinked membranes (S2-CX, S2-CX2, S2-CX3). (c) The ultimate tensile strengths and (d) elongation at break of berberine-containing un-crosslinked membranes (S3-BU, S4-BU) and 1 hour, 2 hour, 3 hour crosslinked membranes (S4-BX, S4-BX2, S4-BX3) (*n* = 4).

Fig. 4a and b display the ultimate tensile strength and elongation at break or break strain of caffeic acid-containing membranes. The tensile strength of un-crosslinked S1-CU is 3.56 MPa, higher than that of the un-crosslinked S2-CU membrane. However, S2-CU exhibits a higher elongation at break, approximately 10%. This demonstrates the common inverse relationship between the ultimate tensile strength and elongation at break, indicating that the inclusion of HTCC reduces the strength of S2-CU, making it more ductile and capable of withstanding substantial deformation before failure. Vapor crosslinking of S2-CU membranes with glutaraldehyde for 1, 2, and 3 hours facilitates the formation of covalent bonds between amino groups on chitosan and HTCC, creating a polymer network structure. This slightly enhances the tensile strength of the crosslinked S2-CU membranes (S2-CX, S2-CX2, S2-CX3), as shown in Fig. 4a. However, this covalent network structure also makes it difficult for polymer chains to undergo large deformation, significantly reducing elongation at break, as shown in Fig. 4b.

The mechanical test results of berberine-containing membranes are presented in Fig. 4c and d. The trends in ultimate tensile strength and elongation at break of berberine-containing membranes mirror those of caffeic acid-containing membranes. The formation of a covalent network structure by glutaraldehyde enhances tensile strength but made the crosslinked membranes brittle. The tensile strength of all the crosslinked HTCC-containing membranes is comparable to that of the electrospun chitosan/alginate/gentamicin nanofiber membrane used as a wound dressing.^56^

Fig. 4 demonstrate minimal changes in the mechanical properties of crosslinked membranes when subjected to vapor crosslinking for more than 1 hour. This observation indicates that most of the free amino groups on HTCC and chitosan formed covalent bonds with the highly reactive glutaraldehyde within the initial 1 hour period. Therefore, to address potential drawbacks such as the formation of brittle membranes due to excessive crosslinking by glutaraldehyde, the optimal crosslinking time is 1 hour for stability test, drug release test, and *in vitro* antibacterial experiments.

### 3.4. Stability test of fibrous membrane

When using electrospun fibrous membranes as wound dressings, they can absorb wound exudate and gradually release their components into the surrounding environment. However, this process can potentially degrade the fibrous structure, especially in aqueous conditions. Therefore, we conduct an *in vitro* stability test on the electrospun membranes to assess their resilience in phosphate-buffered saline solution (PBS).

**Fig. 5:**
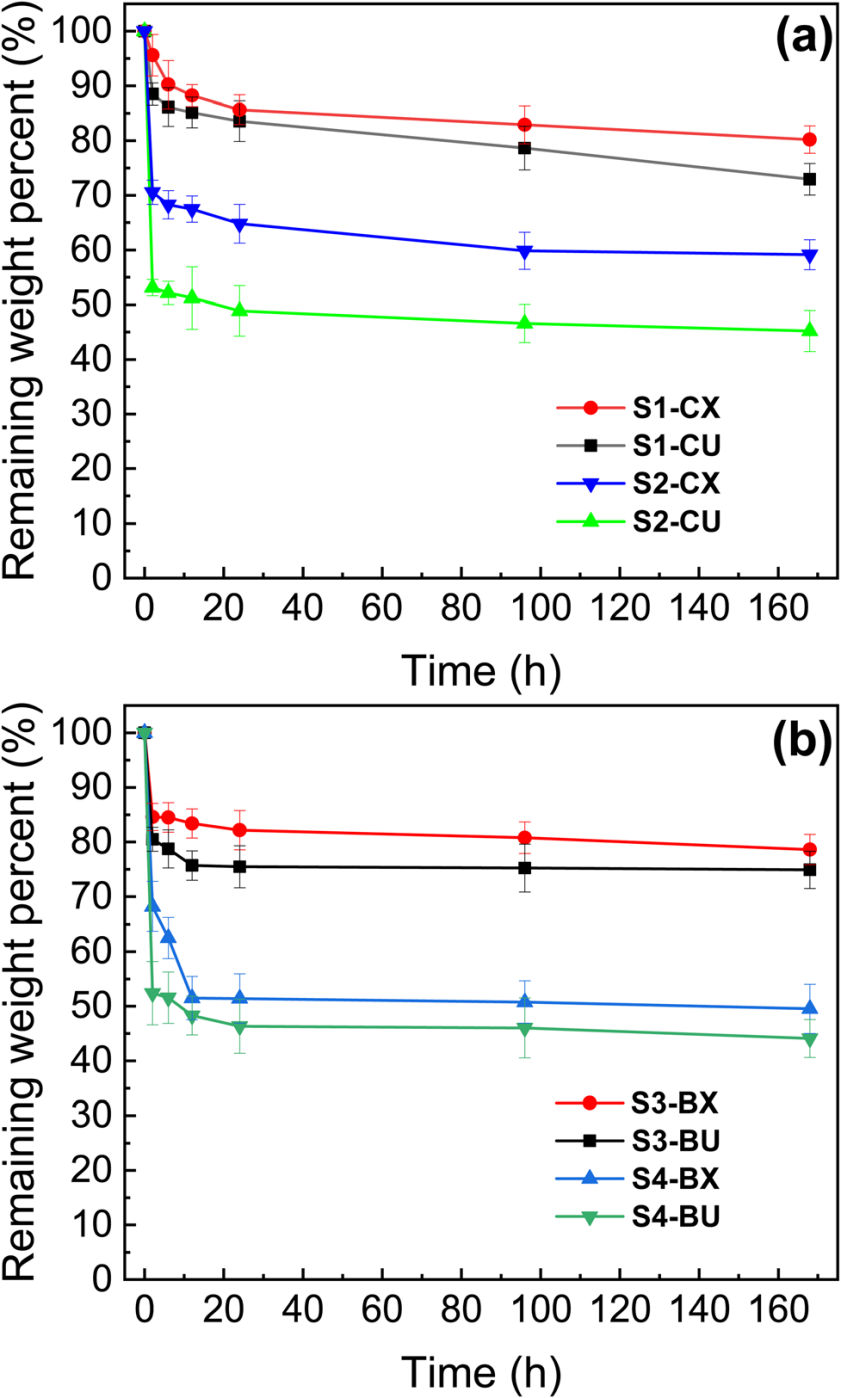
The remaining weight curves of (a) caffeic acid containing membranes, and (b) berberine containing membranes. The samples were collected at 2, 6, 12, 24, 96, 168 hours after immersion in PBS (37 °C, pH 7.4).

Fig. 5a shows the residual weight change curves of electrospun membranes containing caffeic acid over time. In the first 2 hours, membranes containing HTCC (S2-CU and S2-CX) exhibit a more rapid decrease in residual weight compared to those without HTCC (S1-CU and S1-CX). This accelerated weight reduction can be attributed to the water-soluble nature of HTCC, which enhances water absorption and swelling of the fibers, promoting the leaching of water-soluble substances. Consequently, the inclusion of HTCC leads to a higher degree of weight loss in the fibrous membranes. Additionally, a comparison between uncrosslinked membranes (S1-CU and S2-CU) and GA-crosslinked membranes (S1-CX and S2-CX) reveals consistently higher remaining weight percentages for the GA-crosslinked samples at any given time. This suggests that GA enhances the stability of the fibrous membranes by stabilizing water-soluble components such as chitosan, HTCC, and PEO through covalent bonding.

Fig. 5b shows the change in the percentage of remaining weight over time for membranes containing berberine. Membranes with HTCC (S4-BU and S4-BX) consistently show lower remaining weight percentages compared to those without HTCC (S3-BU and S1-CU1), due to the water-soluble nature of HTCC. As a result, GA-crosslinked membranes containing caffeic acid demonstrate significantly improved stability in PBS, exhibiting enhanced wet resistance and durability, which makes these crosslinked membranes suitable for practical use in wound dressings.^57^

### 3.5. Drug release of electrospun membranes

**Fig. 6:**
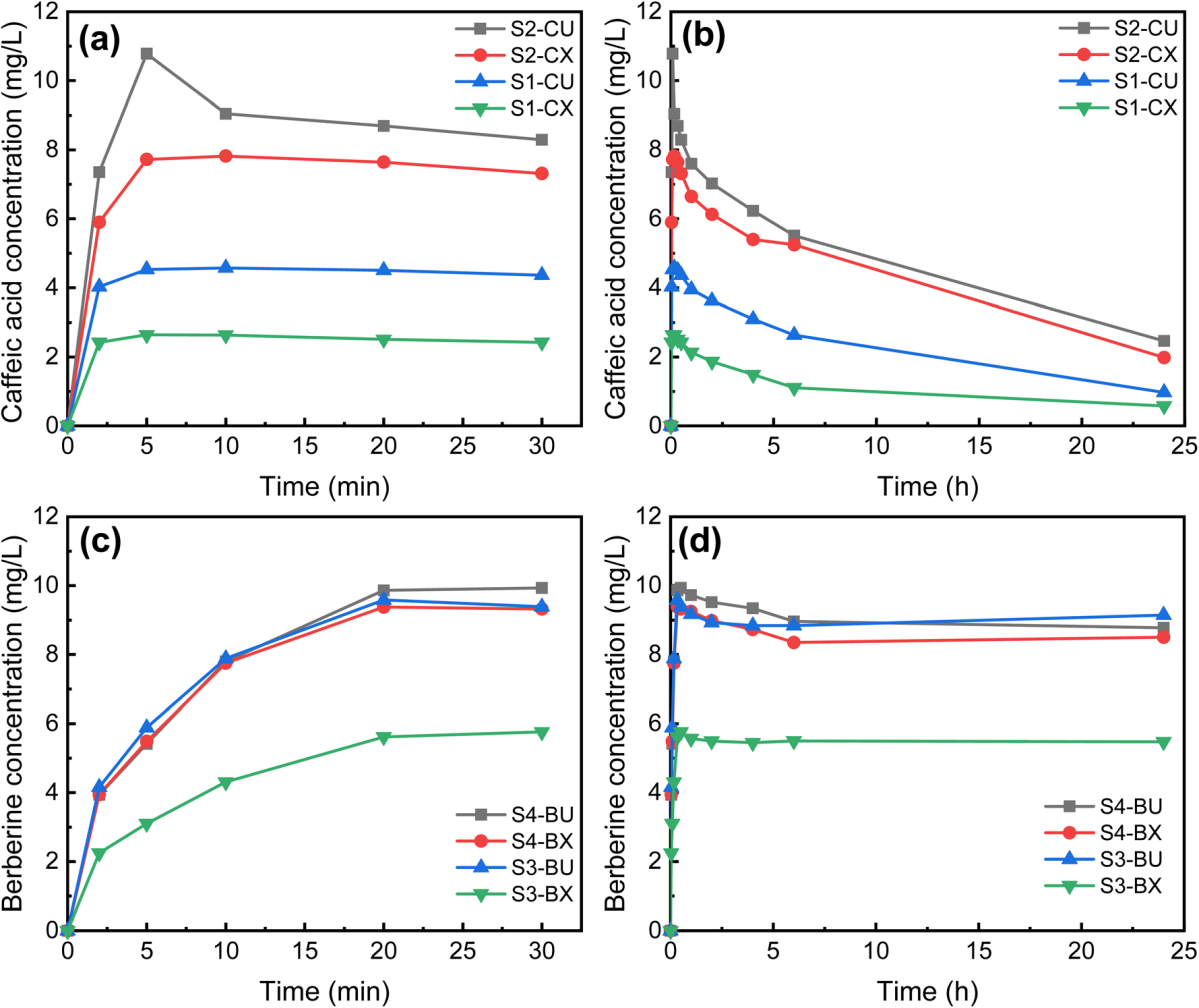
The caffeic acid release profiles of uncrosslinked and crosslinked membranes immersed in 37 °C pH 7.4 PBS within (a) the first 30 minutes and (b) 24 hours. The berberine release profiles of uncrosslinked and crosslinked membranes immersed in 37 °C pH 7.4 PBS for (c) the first 30 minutes and (d) 24 hours.

During the electrospinning process, caffeic acid or berberine, along with the polymers from the formulation, are drawn into fibers and encapsulated within them. As the fibers swell, such as through water absorption, the gradual release of caffeic acid or berberine is facilitated, resulting in a rapid release of the drug. Fig. 6a and c present the short-term (≤30 minutes) release profiles of caffeic acid and berberine chloride, respectively. In Fig. 6a, membranes containing HTCC (S2-CU and S2-CX) exhibit a higher concentration of released caffeic acid compared to those without HTCC (S1-CU and S1-CX). This difference is due to the hydrophilicity of the HTCC polymer chain within the fibers, making it prone to swelling in PBS. Consequently, the swollen HTCC polymer chains lead to a greater release of caffeic acid from the nanofibers.

To achieve controlled drug release, we anticipate that fibrous membranes crosslinked with glutaraldehyde may slow the release of caffeic acid by hindering its diffusion through the crosslinked network structure. As shown in Fig. 6a, the concentrations of caffeic acid released from all four membranes, whether crosslinked or not, quickly reach their peak within the first 5 minutes. However, for S1-CX and S2-CX (crosslinked membranes with or without HTCC), both exhibit reduced polymer swelling due to the crosslinking treatment. Consequently, these membranes release less caffeic acid over time compared to the non-crosslinked membranes (S1-CU and S2-CU). Fig. 6c shows the short-term berberine release profile. All berberine-containing membranes (except for S3-BX) exhibit similar release patterns, regardless of composition. S3-BX, however, shows the lowest drug release, suggesting that the crosslinking network structure within the nanofibers significantly impedes release. Additionally, the absence of water-absorbing HTCC in S3-BX further prevents berberine release by limiting polymer swelling.

Fig. 6b and d show the long-term (up to 24 hours) release profiles of caffeic acid and berberine chloride, respectively. In Fig. 6d, the berberine concentrations remain nearly unchanged after peaking at 0.5 hours. The slight decrease in berberine concentration may be due to the dilution effect from adding pure PBS solution to replace the small amount of drug-release solution withdrawn for concentration measurement. This indicates that berberine exhibits good stability in PBS. In contrast, as shown in Fig. 6b, caffeic acid concentrations reach their maximum at 5 minutes, followed by a significant decrease. This decline is likely due to the degradation of caffeic acid in PBS over time, resulting in a gradual decrease in concentration within 24 hours.^47^ When comparing the drug release profiles of caffeic acid and berberine chloride, it is observed that the maximum concentrations of released berberine chloride are generally higher than those of released caffeic acid. This outcome may be attributed to their significant differences in solubility in acetic acid solution. Caffeic acid is highly soluble in the electrospinning solution, leading to a more uniform distribution throughout the electrospun fibers. In contrast, berberine chloride has lower solubility, causing it to deposit on the fiber surface. As a result, the surface-deposited berberine chloride is more easily released into PBS, likely due to reduced resistance to mass transfer, leading to higher equilibrium concentrations compared to caffeic acid, which remains mostly embedded within the nanofibers. Furthermore, it is evident that both substances are quickly released from the nanofibers, reaching peak concentrations in PBS solution within 30 minutes. This rapid release can be attributed to the high surface area to volume ratio of electrospun membranes, which allows the embedded drugs to dissolve quickly in the solution.^58^ These findings suggest that the chitosan-based composite nanofibers produced in this study hold promise as antibacterial wound dressings, as their ability to rapidly release drugs may quickly inhibit bacterial growth.

### 3.6. Antibacterial test

**Fig. 7:**
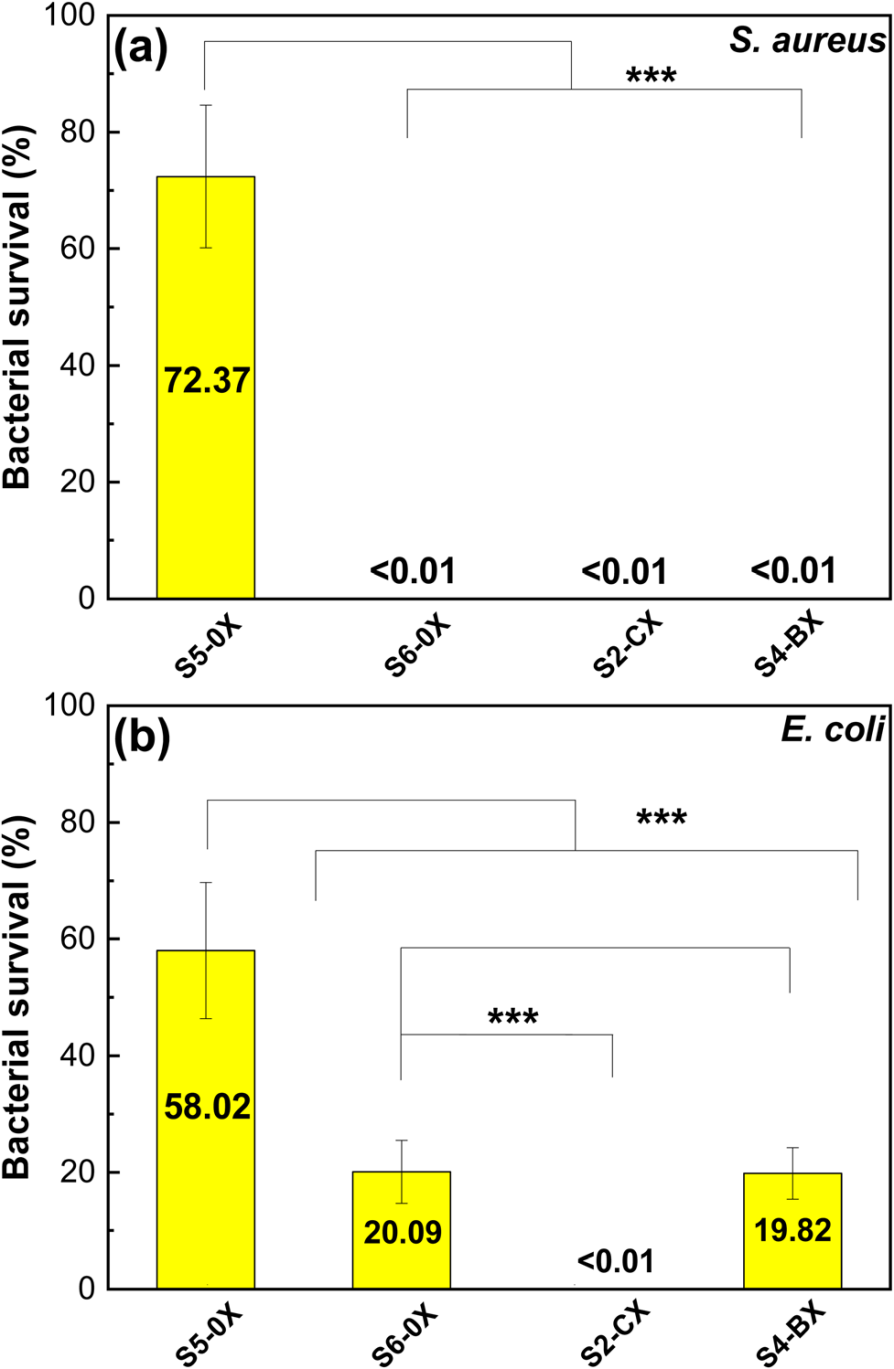
(a) Antibacterial growth test for *S. aureus* cultivated with S5-0X, S6-0X, S2-CX and S4-BX membranes (*n* = 6). (b) Antibacterial growth test for *E. coli* cultivated with S5-0X, S6-0X, S2-CX and S4-BX membranes (*n* = 9) (***p < 0.001 by Student's *t*-test).

To investigate the antibacterial effectiveness of the produced membranes, we tested the crosslinked chitosan membrane, as well as HTCC–chitosan membranes with and without caffeic acid or berberine chloride, for their ability to hinder the growth of both *S. aureus* (Gram-positive) and *E. coli* (Gram-negative) bacteria using the antibacterial assay method following the procedure outlined in Section 2.9. The percentage of bacterial survival for each type of membranes is determined using eqn (3). A lower percentage of bacterial survival indicates greater antibacterial efficacy. Fig. 7a depicts the results of the antibacterial assessment against *S. aureus*. The bacterial survival ratio for the S5-0X is 72.37%.

Conversely, the bacterial survival ratio for the other three types of membranes is nearly 0%, indicating extremely high antibacterial efficacy. The significant difference in antibacterial activity between S5-0X and the other three membranes stems from chitosan being the sole active antibacterial component within S5-0X. However, during the antibacterial tests conducted in a neutral aqueous environment, the proportion of protonated amino groups of chitosan is relatively low, thus limiting the antibacterial effectiveness of S5-0X. On the contrary, the other three types of membranes exhibit remarkably high antibacterial activity against *S. aureus*. This is likely due to the significant role played by HTCC in the antibacterial mechanism. In neutral pH, the polycationic HTCC can be attracted to the negatively charged cell wall of *S. aureus* through electrostatic interaction. The attached HTCC polymer chains may weaken the barrier function of the cell wall, leading to the leakage of intracellular components and ultimately resulting in cellular lysis.^9,59^ Therefore, all three membranes containing HTCC demonstrate excellent antibacterial efficacy, while the S5-0X membrane is less effective. Although caffeic acid and berberine chloride may have also contributed to inhibiting bacterial growth, their individual antibacterial effectiveness against *S. aureus* cannot be conclusively determined from the experimental results shown in Fig. 7a.

Fig. 7b displays the outcomes of antibacterial assessments against *E. coli*. It is evident that the survival ratio of *E. coli* on both S5-0X and S6-0X membranes are higher compared to those of *S. aureus*. This difference can be attributed to the fact that *E. coli* is a Gram-negative bacterium with an outer membrane composed of lipopolysaccharides, which encloses the inner negatively charged cell membrane.^60^ This outer membrane acts as a barrier, preventing further damage to the cell membrane by chitosan and HTCC.^61^ Consequently, despite the presence of HTCC within the S6-0X membrane, the survival ratio of *E. coli* still reaches 20.1%. However, the S2-CX membrane containing caffeic acid exhibits remarkable antibacterial activity against *E. coli*, with the survival percentage of *E. coli* nearing 0% after 12 hour incubation. Caffeic acid likely inhibits the function of efflux pumps on the bacteria's surface, thereby exerting antibacterial effects.^62^ In contrast, the survival ratio of *E. coli* on the S4-BX membrane containing berberine chloride is similar to that of the S6-0X membrane, indicating that berberine chloride possessed minimal antibacterial activity against *E. coli*. This observation suggests that although berberine chloride is a promising antibacterial agent, the concentration of berberine released from S4-BX membranes does not reach an effective inhibitory level against *E. coli*.

The findings illustrated in Fig. 7 affirm that the inclusion of caffeic acid in the S2-CX membrane significantly enhances the antibacterial efficacy of HTCC. Therefore, the S2-CX membrane exhibited outstanding antibacterial activity against both *S. aureus* and *E. coli*. Consequently, it is reasonable to anticipate that the S2-CX membrane holds considerable promise for applications related to antibacterial wound dressings.

## 4. Conclusion

In this study, we successfully fabricated antibacterial nanofibrous membranes using chitosan, quaternized chitosan and either caffeic acid or berberine chloride through electrospinning. The crosslinked membranes demonstrated tensile strengths ranging from 2.7 to 3.0 MPa, comparable to other chitosan-based nanofibers used in wound dressing application. Stability test revealed improved tolerance to aqueous environments for crosslinked membranes, making them advantageous for use in moist wound care. Drug release tests showed rapid release of both caffeic acid and berberine chloride from the nanofibers, potentially promoting swift inhibition of bacterial growth in aqueous conditions. Antibacterial tests demonstrated that the S2-CX membrane, containing caffeic acid, exhibited outstanding antibacterial activity against both *S. aureus* and *E. coli*, with a near 0% survival rate after a 20 hour incubation. S4-BX also showed antibacterial activity, with near 0% survival of *S. aureus* and 19.82% survival of *E. coli*. The superior antibacterial efficacy of S2-CX against *E. coli*, compared to S4-BX, is likely due to caffeic acid's effectiveness in inhibiting the efflux pump function of *E. coli*, preventing the removal of antibacterial toxins from its cytoplasm. In summary, the S2-CX nanofiber membrane demonstrates suitable tensile strength and enhanced antibacterial activity, positioning it as a promising candidate for advanced antibacterial wound dressings and drug delivery applications. Further research into the biocompatibility of these membranes will be conducted in the near future.

## Data availability

The raw data supporting the conclusions of this article will be made available by the authors on request.

## Author contributions

All authors have read and agreed to the final version of the manuscript. P. H. C., C. C. H., C. M. H.: data curation; formal analysis; visualization; writing – original draft preparation. Z. Y. W.: *in vitro* antibacterial tests; formal analysis. C. T. H.: visualization; writing – original draft preparation. C. M. L.: methodology. Y. C. C.: methodology; resources; writing – original draft preparation; supervision. S. C. C., H. J. H., C. A. D.: methodology; conceptualization; writing – review & editing; funding acquisition; project administration; resources; supervision.

## Conflicts of interest

The authors declare no conflicts of interest.

## Supplementary Material

RA-014-D4RA05114A-s001

RA-014-D4RA05114A-s002

RA-014-D4RA05114A-s003

RA-014-D4RA05114A-s004

RA-014-D4RA05114A-s005
