# Supplementary figures and images for: Enhancement of antibacterial activity in electrospun fibrous membranes based on quaternized chitosan with caffeic acid and berberine chloride for wound dressing applications
Source: RSC Adv. 2024 Oct 30;14(47):34756–68. doi: 10.1039/d4ra05114a (PMC11526035; doi:10.1039/d4ra05114a)

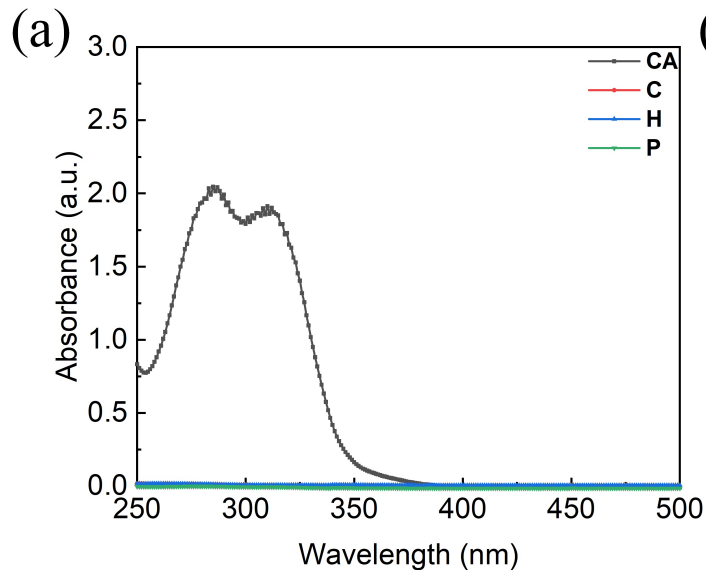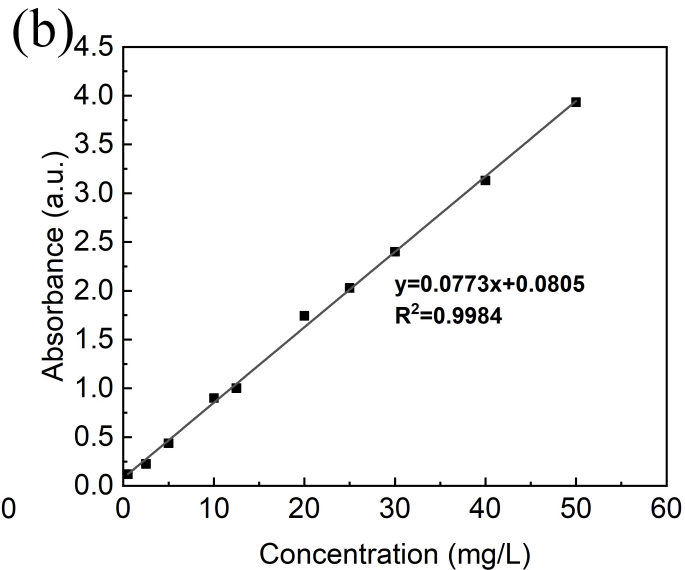

Supplement: RA-014-D4RA05114A-s002 [file RA-014-D4RA05114A-s002.pdf]

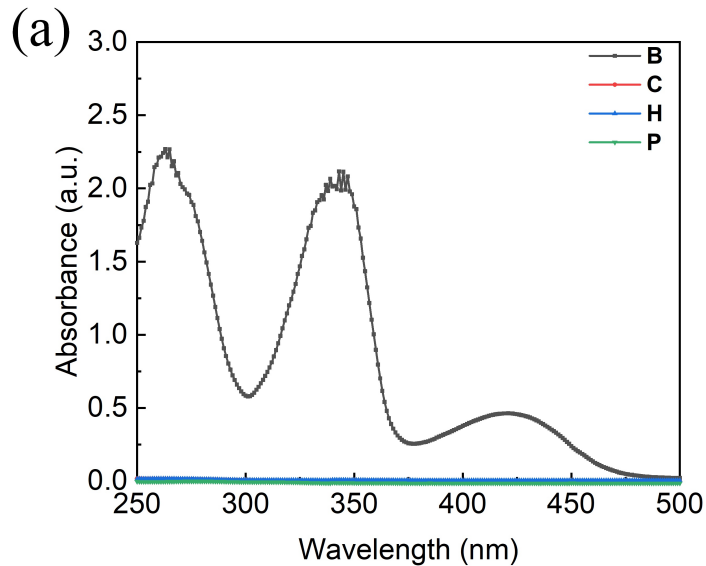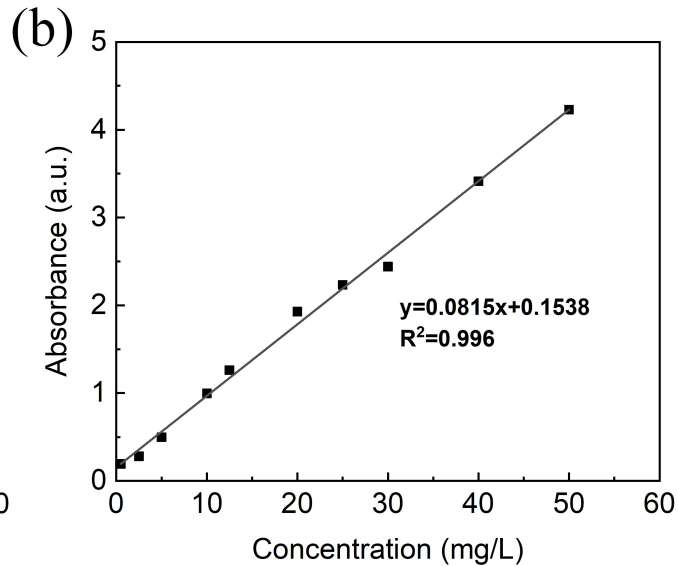

Supplement: RA-014-D4RA05114A-s003 [file RA-014-D4RA05114A-s003.pdf]

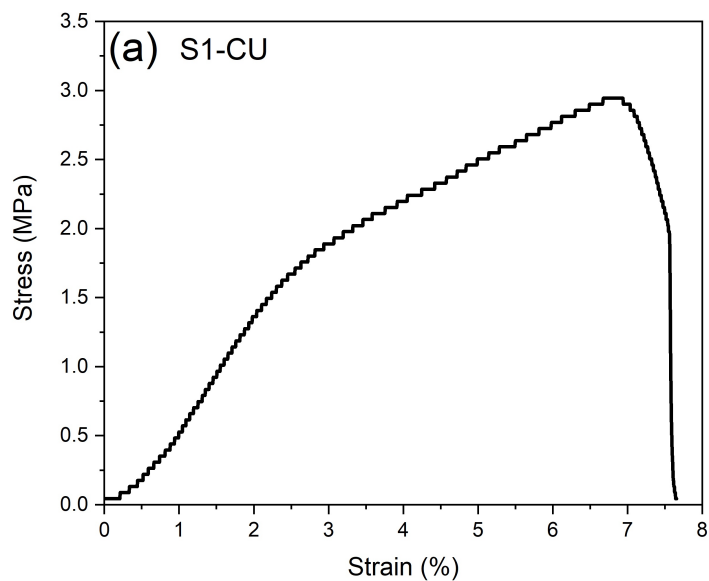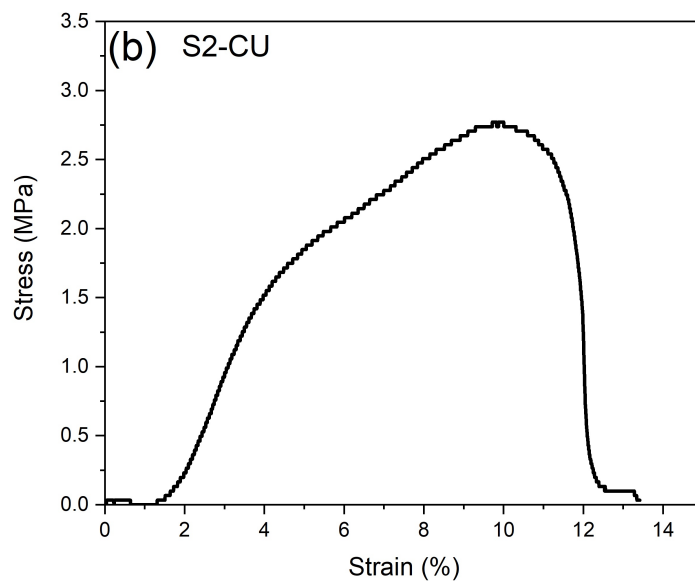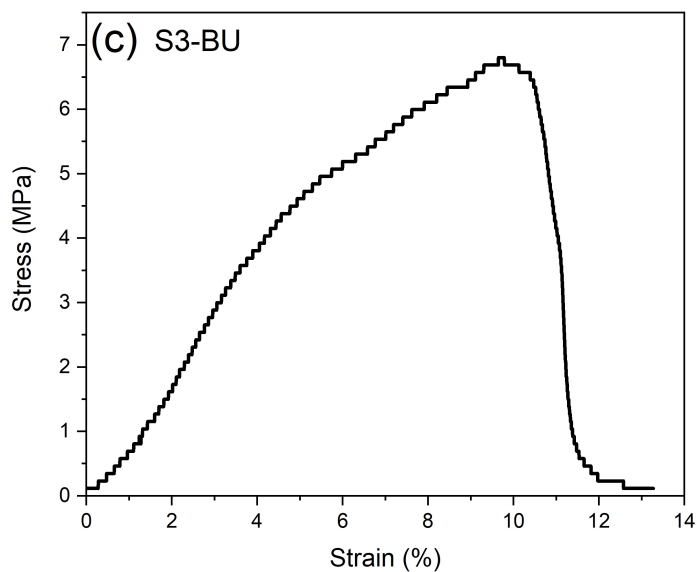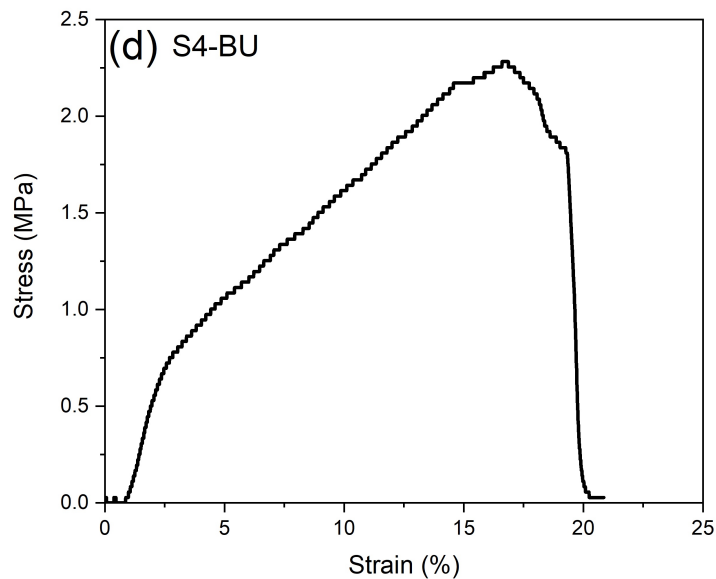

Supplement: RA-014-D4RA05114A-s004 [file RA-014-D4RA05114A-s004.pdf]
