# Supplementary material for: Enhancement of antibacterial activity in electrospun fibrous membranes based on quaternized chitosan with caffeic acid and berberine chloride for wound dressing applications
Source: RSC Adv. 2024 Oct 30;14(47):34756–68. doi: 10.1039/d4ra05114a (PMC11526035; doi:10.1039/d4ra05114a)
Supplement: RA-014-D4RA05114A-s005 [file RA-014-D4RA05114A-s005.pdf]

Supplementary Information (SI) for RSC Advances.
This journal is © The Royal Society of Chemistry 2024

Supplementary information

Enhancement of Antibacterial Activity in Electrospun Fibrous Membranes Based on Natural Compounds for Wound Dressing Applications

Po-Hsun Chiu[1], Zhao-Yi Wu[2], Chih-Chin Hsu[1], Yung-Chi Chang[2], Chang-Ming Huang[1], Cheng-Ti Hu[1], Che-Min Lin[1], Shin C. Chang[2], Hsyue-Jen Hsieh[1]*, Chi-An Dai[1]*

[1]Department of Chemical Engineering, National Taiwan University, Taipei 10617, Taiwan

[2]Graduate Institute of Microbiology, College of Medicine, National Taiwan University, Taipei 10051, Taiwan

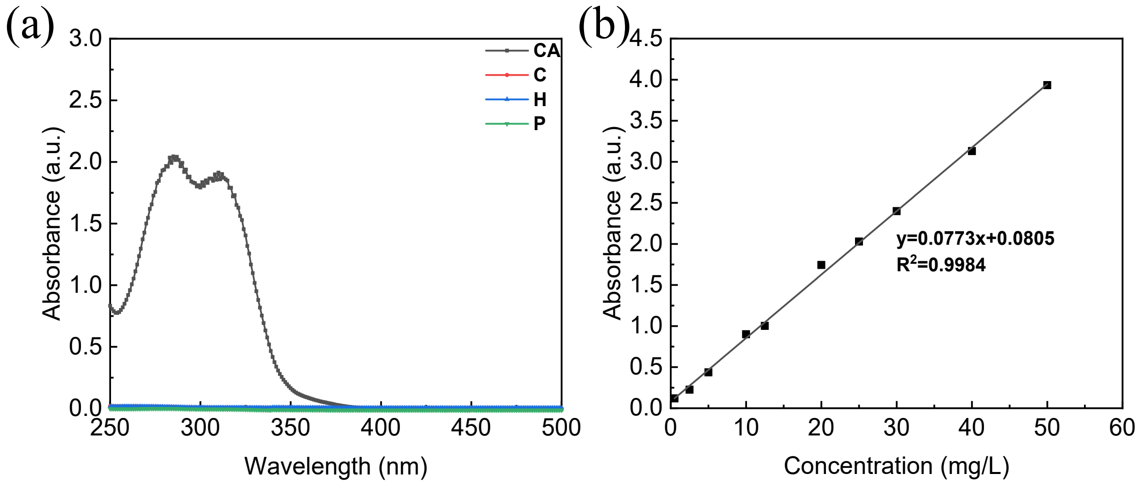

Fig. S1 (a) 250 to 500 nm UV/VIS spectra of caffeic acid (CA), chitosan (C), HTCC (H) and polyethylene oxide (P) dissolved in pH 7.4 PBS with a concentration of 25 mg/L; (b) Calibration line of caffeic acid measured at 283 nm ranging from 0.5 – 50 ppm.

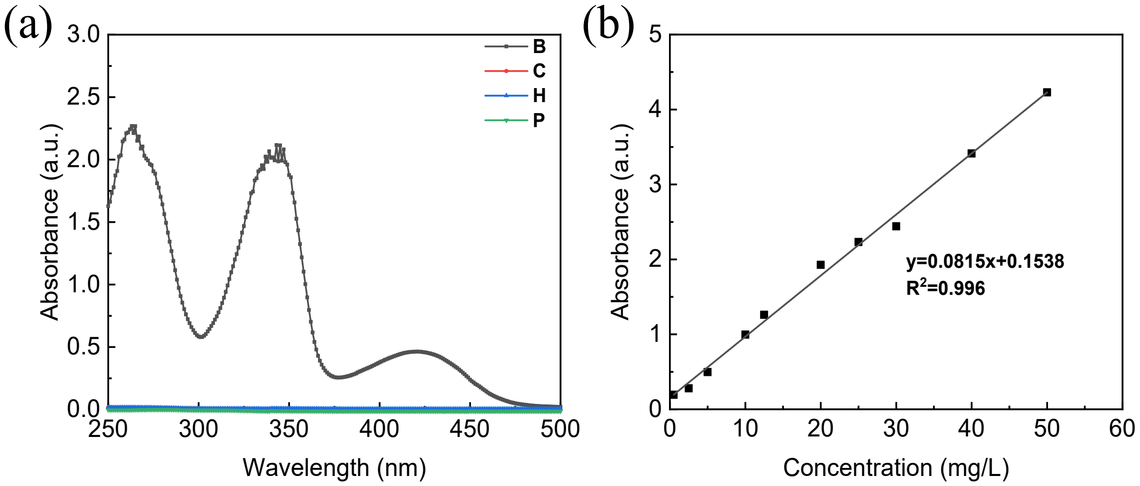

Fig. S2 (a) 250 to 500 nm UV/VIS spectra of berberine (B), chitosan (C), HTCC (H) and polyethylene oxide (P) dissolved in pH 7.4 PBS with a concentration of 25 mg/L; (b) Calibration line of berberine measured at 260 nm ranging from 0.5 – 50 ppm.

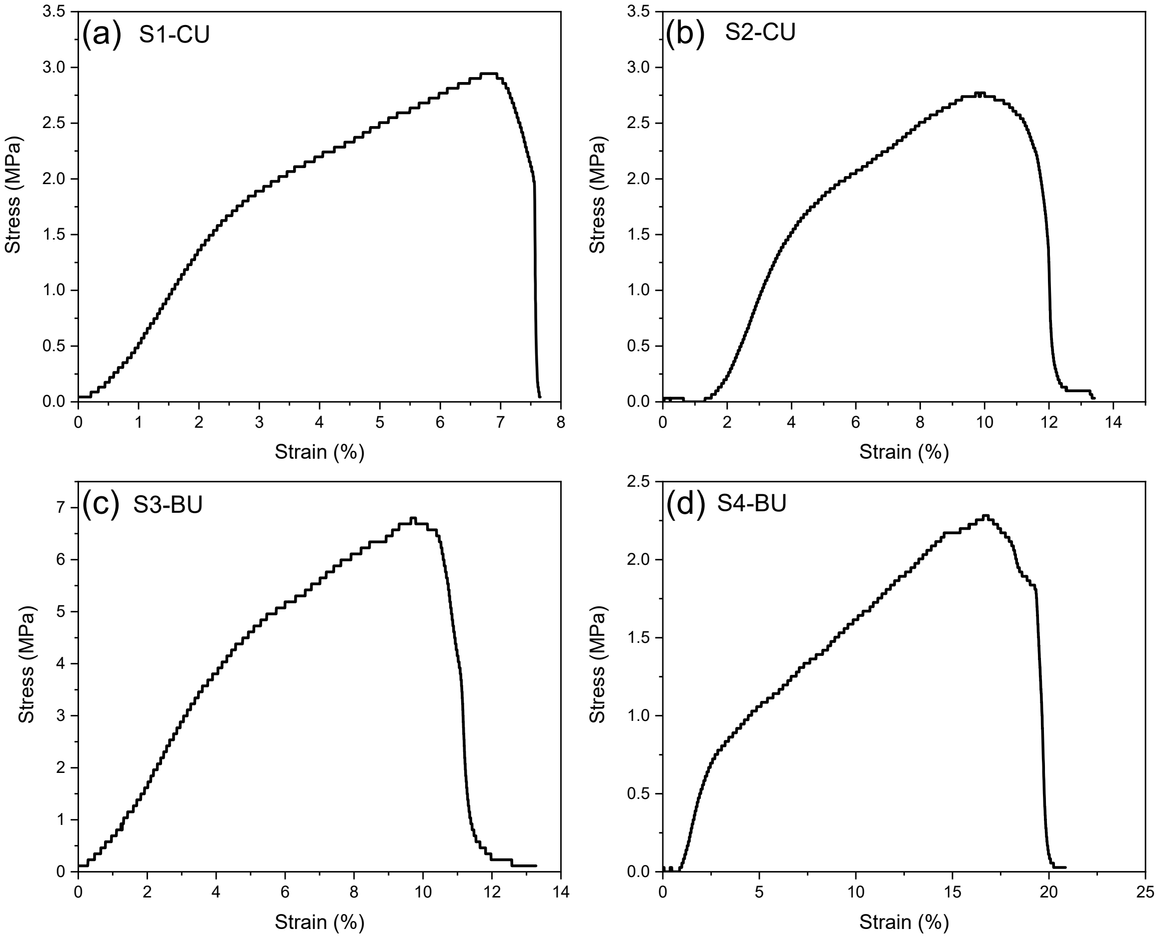

Fig. S3 Stress-strain curves of **(a)** S1-CU, **(b)** S2-CU, **(c)** S3-BU and **(d)** S4-BU electrospun nanofibers.
